# A multidimensional in-depth analysis of postoperative pain after PLIF in patients with degenerative lumbar spine disease

**DOI:** 10.3389/fmed.2025.1691596

**Published:** 2025-12-11

**Authors:** Ting Tang, Xiao Sun, Haifu Sun, Yonggang Li, Yimeng Wang, Dan Chen

**Affiliations:** Department of Orthopedics, The First Affiliated Hospital of Soochow University, Suzhou, China

**Keywords:** posterior lumbar interbody fusion (PLIF), degenerative lumbar spine disease, postoperative pain, paraspinal muscle indices (PMI, MMI), early exercise

## Abstract

**Purpose:**

To comprehensively investigate multifactorial influences on postoperative pain in patients with degenerative lumbar spine disease undergoing posterior lumbar interbody fusion (PLIF), thereby guiding targeted rehabilitation.

**Patients and methods:**

We reviewed 316 patients (age >40) who underwent PLIF from January 2022 to May 2024. Based on postoperative Numeric Rating Scale (NRS) scores and pain duration, they were divided into a non-pain group (210 cases) and a pain group (106 cases). We assessed paraspinal muscle indices (PMI, MMI), Self-Rating Anxiety Scale (SAS), postoperative exercise duration, surgical segments, drainage removal time, and mannitol usage. Univariate and multivariate logistic regression analyses were conducted.

**Results:**

No significant differences were found in age, BMI, education, surgical duration, drainage time, disease duration, time to first ambulation, or mannitol usage (*P* > 0.05). However, gender, SAS scores, surgical segments, drainage volume at removal, PMI, MMI, average standing time in the first 2 weeks, and duration of straight-leg raise exercises differed significantly (*P* < 0.05). Multivariate analysis identified surgical segment (*P* = 0.008), drainage volume at removal (*P* = 0.008), MMI (*P* < 0.001), average standing time (*P* = 0.010), and straight-leg raise exercise duration (*P* = 0.012) as independent risk factors.

**Conclusion:**

Paraspinal muscle health and early postoperative exercise are crucial factors influencing PLIF-related postoperative pain. Tailored rehabilitation, enhanced muscle function, and optimized early exercise may reduce postoperative pain and improve outcomes.

## 1 Introduction

With the accelerated pace of global population aging, the incidence of degenerative lumbar spine diseases continues to rise among middle-aged and elderly populations. These pathologies, including lumbar disc herniation, spinal stenosis, intervertebral disc degeneration, segmental instability, and degenerative scoliosis, frequently manifest as low back pain, radiating leg pain, intermittent claudication, and nerve root irritation, thereby exerting a substantial adverse impact on patients' quality of life and daily functioning (1, 2). For patients who do not achieve satisfactory relief with conservative management, surgical intervention has become a pivotal approach to alleviating pain and restoring function. Among various surgical techniques, posterior lumbar interbody fusion (PLIF), a classical and effective procedure, is widely employed in the treatment of degenerative lumbar spine disorders (3, 4). By removing the affected intervertebral disc via a posterior approach and placing an interbody cage supported by pedicle screw fixation, PLIF restores lumbar stability and physiological alignment, creating favorable conditions for eventual intervertebral fusion and adequate neural decompression. A substantial body of evidence has confirmed that PLIF can significantly improve neurological symptoms, mitigate pain, and restore functional status over mid- to long-term follow-up (5, 6).

Despite these favorable longer-term outcomes, a considerable proportion of patients experience pronounced postoperative pain during the early postoperative period. This early postoperative pain not only impedes the patient's early rehabilitation, activity level, and quality of life but may also delay functional exercise and psychological recovery, consequently intensifying anxiety, fear, patient dissatisfaction, and the consumption of healthcare resources. Inadequate postoperative pain control can lead to negative emotions, compromise patient–physician communication, and impede effective care and evaluation of outcomes (7). Previous studies have suggested that the mechanisms underlying postoperative pain are multifactorial, encompassing surgical invasiveness, anesthetic and analgesic strategies, the degree of nerve compression, and individual patient characteristics [e.g., age, sex, body mass index (BMI), comorbidities] (8–10). Nonetheless, these factors alone often fail to sufficiently explain the complexity and multifaceted nature of postoperative pain.

In recent years, growing attention has been directed toward the role of paraspinal muscle structure and function in influencing postoperative pain and the rehabilitation process (11). The paraspinal musculature, especially the multifidus and psoas major muscles, plays a crucial role in maintaining spinal stability, distributing mechanical loads, and controlling spinal motion. Alterations in muscle quality, the extent of fatty infiltration, and functional decline may all affect patients' pain perception and recovery trajectory (12, 13). Previous studies have linked diminished multifidus muscle quality with low back pain, muscle fatigue, and compromised spinal stability (14, 15). Moreover, the posterior surgical approach and soft tissue dissection in PLIF may further aggravate paraspinal muscle injury, thereby contributing to postoperative pain and delayed functional recovery. Quantitative assessment of paraspinal muscle indices via MRI, such as the Psoas Muscle Index (PMI) and Multifidus Muscle Index (MMI), offers an objective evaluation of muscle status and may provide a novel perspective for predicting postoperative pain. Although the measurement of cross-sectional areas or fat infiltration of the multifidus and psoas muscles has been proposed as an indicator of muscle health and a potential target for postoperative rehabilitation strategies, evidence directly substantiating the association between these paraspinal muscle parameters (e.g., PMI, MMI) and early postoperative pain remains limited (16). A comprehensive investigation is warranted to clarify the extent to which these muscle-related factors contribute to short-term pain outcomes.

Beyond local biological factors, psychological determinants of postoperative pain have also garnered increasing attention. Perioperative anxiety, fear, and other negative emotions may amplify subjective pain perception through neuroendocrine and cognitive pathways. The Self-Rating Anxiety Scale (SAS) is commonly employed to quantify patients' anxiety levels, and higher anxiety scores have been correlated with increased postoperative pain intensity. Additionally, postoperative rehabilitation practices, including early mobilization and appropriate functional exercise, are critical for reducing pain, enhancing metabolic circulation, and minimizing local inflammatory accumulation. Nonetheless, disparities in rehabilitation intensity and timing may trigger local stress and discomfort, thereby negating potential benefits.

In summary, early postoperative pain after PLIF represents a complex phenomenon resulting from the interplay of surgical variables (such as the number of fused segments and volume of drainage), paraspinal muscle parameters (PMI, MMI), psychological factors (SAS anxiety scores), and behavioral aspects (postoperative exercise duration, timing of drain removal, medication regimens). While previous studies have frequently focused on isolated factors, our investigation adopts a more holistic approach. By incorporating univariate and multivariate logistic regression analyses to integrate surgical parameters, muscle indices, psychological status, and rehabilitation timelines, we aim to elucidate the multifactorial mechanisms underlying short-term postoperative pain following PLIF. We anticipate that these findings will enrich clinical decision-making and inform individualized perioperative interventions, ultimately improving patient satisfaction, facilitating early rehabilitation, and offering new insights into enhancing the quality of life for patients with degenerative lumbar spine disease.

## 2 Methods

### 2.1 Research design

This study is a retrospective case-control analysis, approved by the relevant ethics committee. The study population comprised 316 patients who underwent posterior lumbar interbody fusion (PLIF) for degenerative lumbar spine disease at our institution between January 2022 and May 2024. All patients were followed up for 3 months postoperatively through daily telephone calls or completion of questionnaires to assess postoperative pain and associated factors. Inclusion criteria: (1) patients aged over 40 years, diagnosed with degenerative lumbar spine disease (such as lumbar disc herniation, lumbar spinal stenosis, etc.) based on lumbar spine MRI, (2) patients who underwent PLIF surgery, with available imaging data and complete postoperative follow-up information. Exclusion criteria: (1) history of lumbar spine surgery, presence of bone tumors, ankylosing spondylitis, rheumatoid arthritis, or secondary osteoporosis, (2) use of corticosteroid treatment or other systemic diseases that affect bone metabolism.

### 2.2 Group indicators

Using the Numeric Rating Scale (NRS) during postoperative follow-up, we assessed the functional improvement and pain relief in patients from baseline to 1 week postoperatively. The NRS evaluates pain intensity over the past week, with scores ranging from 0 (no pain) to 10 (worst imaginable pain) (10). Based on these results, patients were divided into two groups: Group A (non-pain group) included 210 patients with an NRS score of less than 3 at 1 week postoperatively. Group B (pain group) included 106 patients with an NRS score of 3 or higher at 1 week postoperatively. We analyzed the distribution differences between the two groups regarding demographic data, psychological status, imaging findings, postoperative exercise, and prognosis for statistical significance.

### 2.3 Assessment parameters

**Figure 1:**
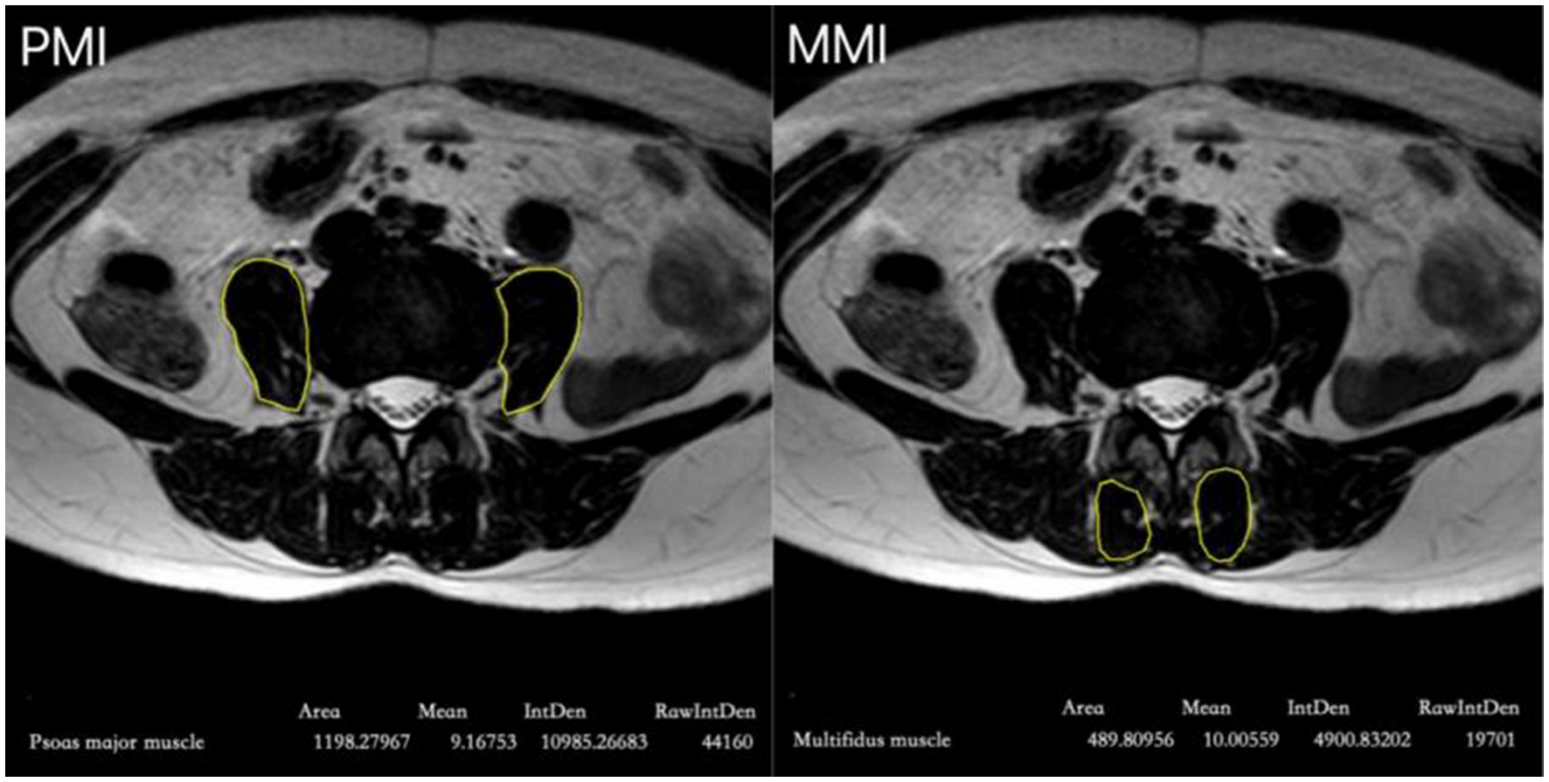
Measurement of PMI and MMI at the level of the superior endplate of the L4 vertebra on MR T2 imaging. Using medical imaging software (ImageJ), we defined regions of interest (ROI) in the psoas and multifidus muscle areas at the superior endplate of the fourth lumbar vertebra in the patient, and measured the muscle areas. The results were 11.98 cm^2^ for the psoas muscle and 4.90 cm2 for the multifidus muscle. The psoas muscle index (PMI) and multifidus muscle index (MMI) for this patient were calculated to be 4.68 and 1.91, respectively.

This study employed multiple assessment parameters to comprehensively evaluate postoperative pain and recovery. The Self-Rating Anxiety Scale (SAS) was used to assess the patients' psychological state and anxiety levels. Following standard operating procedures, we calculated index scores as raw scores × 1.25 (range 25–100) and categorized anxiety levels as follows: < 50 = no anxiety; 50–59 = mild; 60–69 moderate; ≥70 severe. Categorized anxiety severity into four levels (0–3) based on standardized scores: no anxiety or mild anxiety, moderate anxiety, or severe anxiety (17). The health status of the paraspinal muscles was evaluated using the psoas muscle index (PMI) and multifidus muscle index (MMI). Using ImageJ software, the psoas and multifidus muscle areas were measured at the L4 level on MR T2-weighted images (Figure 1). The PMI and MMI were calculated by dividing the muscle area by the square of the patient's height, with the units in square centimeters per square meter (cm^2^/m^2^). The time of the patient's first postoperative ambulation was recorded as an important indicator of early postoperative exercise. Additionally, the patients' rehabilitation plans and actual exercise were assessed by recording the daily average standing time and duration of straight leg raise exercises within the first 2 weeks postoperatively.

### 2.4 Statistical analysis

Statistical analysis was performed using SPSS 27.0 (IBM, Armonk, New York, USA). For normally distributed measurement data, results are expressed as mean ± standard deviation (Mean ± SD). Independent samples *t*-tests were used to compare the quantitative data differences between the two groups of patients, including age, body mass index (BMI), surgical duration, drainage time, psoas muscle index (PMI), multifidus muscle index (MMI), and the average duration of straight leg raise exercises during the first 2 weeks postoperatively. For qualitative data, such as gender, education level, SAS score, drainage volume at extubation, surgical segment, time to ambulation, average daily standing time within the first 2 weeks postoperatively, and the number of days of postoperative mannitol use, chi-square tests were used for intergroup comparisons. Differences between variables and their impact on early postoperative pain were visualized. To further analyse the potential effects of various factors on postoperative pain, univariate and multivariate logistic regression analyses were conducted to assess the independent contribution of each variable to postoperative pain. The corresponding odds ratios (OR) and 95% confidence intervals (CI) were calculated. All statistical results were rigorously tested to ensure the reliability and accuracy of the conclusions, with a *P*-value < 0.05 considered statistically significant.

## 3 Results

### 3.1 Patient characteristics

This study included 316 patients with degenerative lumbar spine disease who underwent posterior lumbar interbody fusion (PLIF), with 55.06% of the patients being female. The age range was from 41 to 82 years, with an average age of 64.5 years. The patients were divided into Group A (non-pain group, 210 patients) and Group B (pain group, 106 patients). No significant differences were found between the two groups in terms of age, BMI, education level, surgical duration, postoperative drainage time, disease duration, time to first ambulation, and mannitol use (*P* > 0.05). However, significant differences were observed between the two groups in terms of gender, SAS scores, surgical segments, drainage volume at extubation, PMI, MMI, average daily standing time during the first 2 weeks, and the duration of straight leg raise exercises (*P* < 0.05). The detailed baseline characteristics, clinical data, and postoperative follow-up results of the patients are shown in Table 1.

**Table 1:** Comparison of general data between the PLIF non-pain group (Group A) and the short-term pain group (Group B) patients.

**Influencing factor**	**Group A (*n* = 210)**	**Group B (*n* = 106)**	***P-*value**
**Gender (%)**			
Male	104 (49.52)	38 (35.85)	0.021
Female	106 (50.48)	68 (64.15)	
Age (mean, SD)	59.33 (13.09)	59.41 (11.41)	0.960
BMI (mean, SD)	24.49 (2.13)	24.25 (2.32)	0.361
**Education (%)**			
Junior high school education	76 (36.19)	37 (34.91)	0.490
High school and undergraduate programs	110 (52.38)	61 (57.54)	
Graduate degree	24 (11.43)	8 (7.55)	
**SAS (%)**			
0	173 (82.38)	70 (66.04)	0.014
1	26 (12.38)	23 (21.70)	
2	8 (3.81)	10 (9.43)	
3	3 (1.43)	3 (2.83)	
**Surgical segment (%)**			
1	164 (78.10)	67 (63.21)	0.012
2	37 (17.61)	28 (26.41)	
3	9 (4.29)	11 (10.38)	
Surgical duration (mean, SD)	2.49 (0.52)	2.59 (0.72)	0.217
Drainage time (mean, SD)	2.41 (0.82)	2.52 (0.77)	0.276
**Drainage volume (%)**			
< 50 ml	175 (83.33)	76 (71.70)	0.016
≥50 ml	35 (16.67)	30 (28.30)	
Duration of illness	23.27 (13.01)	21.47 (12.22)	0.238
PMI (mean, SD)	5.70 (3.09)	4.59 (2.85)	0.002
MMI (mean, SD)	3.51 (0.61)	2.96 (0.52)	< 0.001^*^
**Time to ambulation (%)**			
< 4 days	107 (50.95)	52 (49.06)	0.750
≥4 days	103 (49.05)	54 (50.94)	
**Average daily standing time (2 weeks) (%)**			
< 90 min	160 (76.19)	65 (61.32)	0.006
≥90 min	50 (23.81)	41 (38.68)	
**Mannitol administration (%)**			
< 4 days	111 (52.86)	61 (57.55)	0.429
≥4 days	99 (47.14)	45 (42.45)	
Straight leg raise exercise (2 weeks) (mean, SD)	1.04 (0.69)	0.81 (0.57)	0.003

SD, standard deviation; SAS, Self-Rating Anxiety Scale; PMI, Psoas Muscle Index; MMI, Multifidus Muscle Index. ^*^Represents P < 0.001.

### 3.2 Data visualization

**Figure 2:**
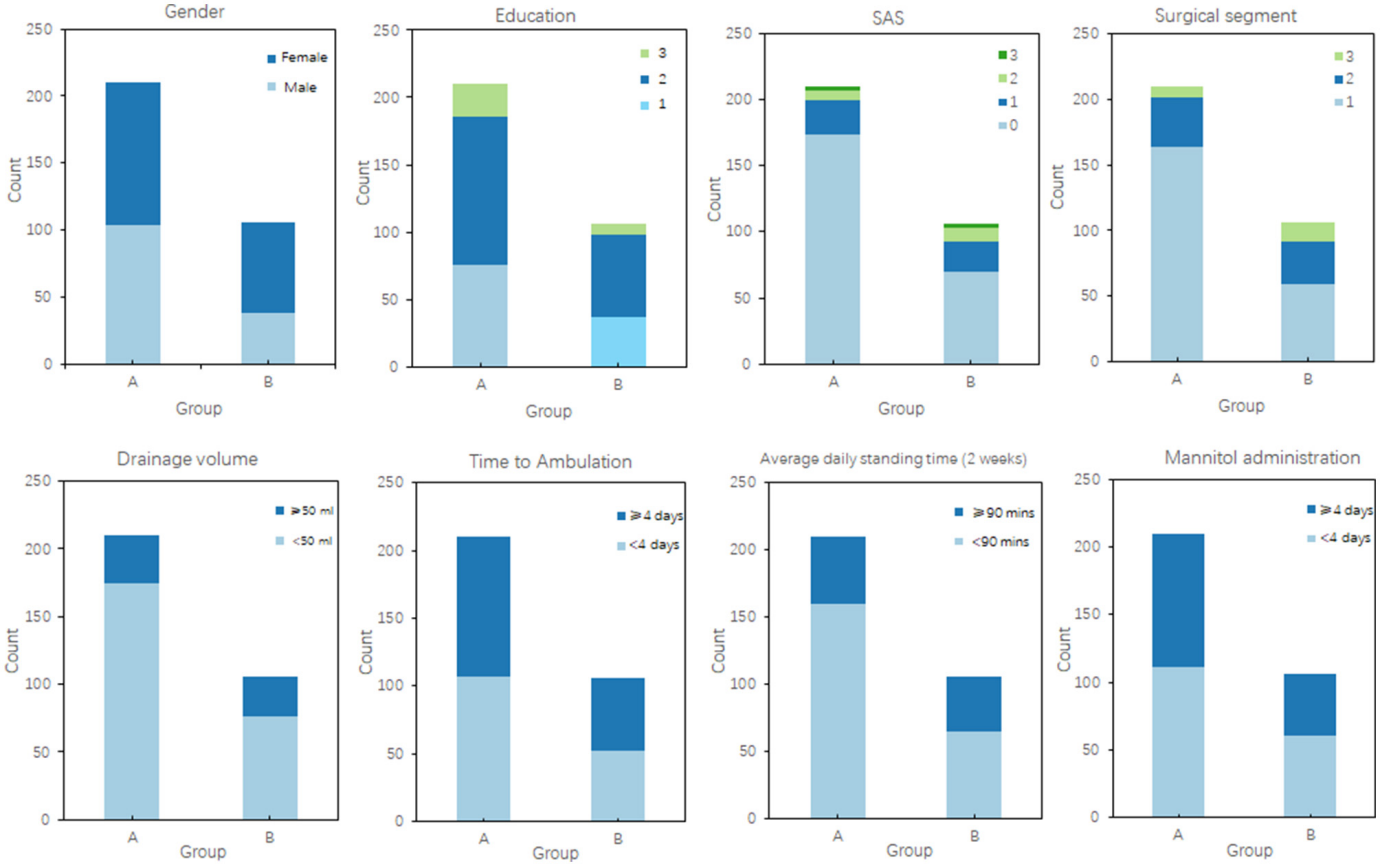
Bar chart comparison of qualitative data between groups. This figure shows the distribution of several categorical variables—Gender, Education level, SAS score, surgical segment, drainage volume at extubation, time to first ambulation, average daily standing time in the first 2 weeks postoperatively, and mannitol use. The bar plots compare the count of each category within these variables, providing a clear visual comparison of the frequency distributions between the two groups.

**Figure 3:**
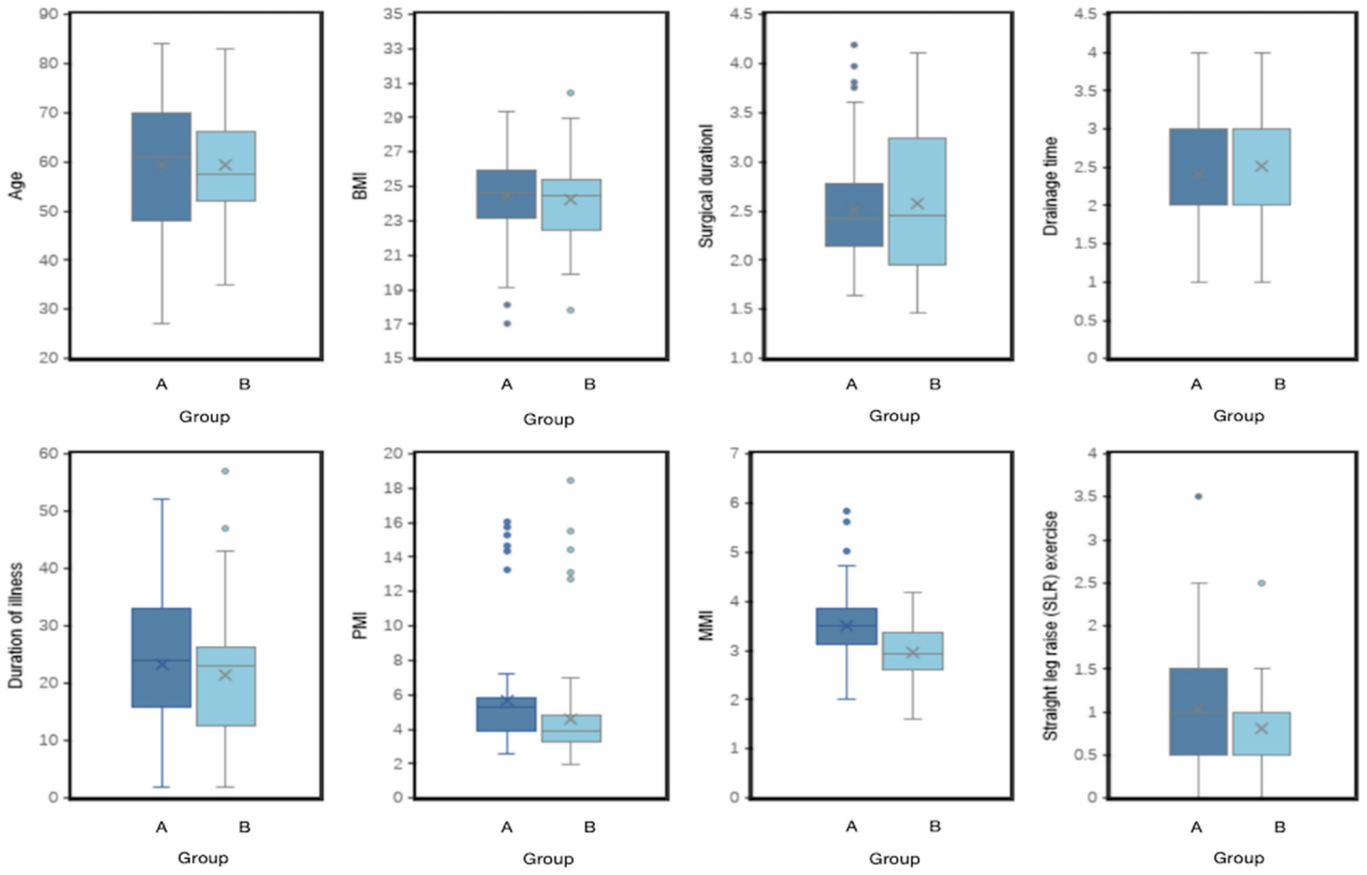
Box plot comparison of continuous variables by Groups. This figure shows the distribution of continuous variables (such as age, PMI, MMI, and duration of straight leg raise exercises) in Group A and Group B. Using box plots and scatter plots, it displays the median, quartiles, and outliers of each variable in different groups, providing a visual comparison of the differences between these variables.

To visually illustrate the relationship between clinical variables and postoperative pain duration, we employed bar charts, box plots, and correlation matrices for analysis. The bar chart displayed the differences in qualitative data between the non-pain group and the early pain group (Figure 2). Patients in Group A (non-pain group) generally had higher education levels and showed lower anxiety levels, as indicated by the SAS scores. In contrast, patients in the pain group had significantly higher drainage volumes at extubation and longer daily standing times. Additionally, the box plot clearly demonstrated the differences in quantitative data between the two groups (Figure 3). The paraspinal muscle-related indices (PMI and MMI) and the duration of straight leg raise exercises were significantly higher in Group A compared to Group B, suggesting that the level of paraspinal muscle health and postoperative exercise are related to pain levels following PLIF surgery.

**Figure 4:**
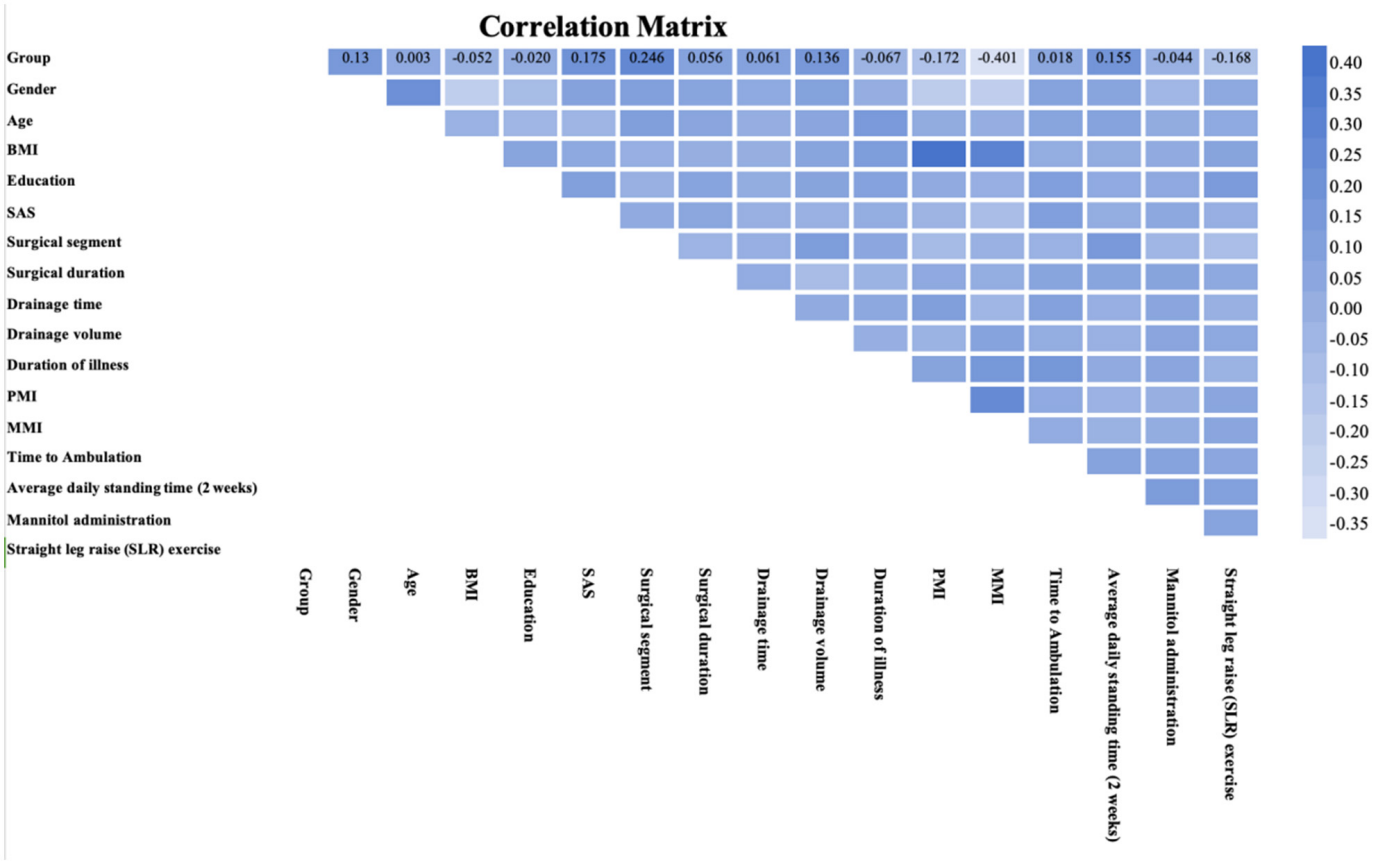
Correlation matrix between each variable and the outcome. This heatmap visualizes the correlation matrix of multiple variables, displaying the strength and direction of linear relationships between them. The color scale indicates the correlation coefficient values, with darker shades representing stronger positive or negative correlations.

In exploring the multidimensional factors influencing postoperative pain management, the correlation matrix provided a powerful tool for quantifying the relationships between various clinical variables and postoperative pain duration (Figure 4). The multifidus muscle index (MMI) showed the strongest negative correlation with postoperative pain, with a correlation coefficient of −0.401, indicating that lower MMI may be associated with more severe postoperative pain. Additionally, factors such as the patients' psychological state, psoas muscle index (PMI), average daily standing time, and the duration of straight leg raise exercises were also strongly correlated with pain duration after PLIF surgery.

### 3.3 Univariate and multivariate analysis

Univariate analysis showed significant differences between the non-pain group and the early pain group in terms of gender, SAS score, surgical segment, drainage volume at extubation, PMI, MMI, average daily standing time (2 weeks), and duration of straight leg raise exercise (2 weeks; *P* < 0.05). Using the NRS score ≥3 at 1 week postoperatively as the dependent variable (No = 0, Yes = 1), the binary logistic regression model was constructed with the variables that were statistically significant in the univariate analysis as independent variables. The Hosmer–Lemeshow (H–L) test was used to assess the goodness-of-fit of the model, with *P* > 0.05 indicating no statistically significant difference and thus a satisfactory model fit. The H–L test for the risk prediction model showed *X*^2^ = 12.539, *P* = 0.129, indicating that the regression model in this study had a good fit. Multivariate analysis revealed that surgical segment (*P* = 0.008), drainage volume at extubation (*P* = 0.008), MMI (*P* < 0.001), average daily standing time (2 weeks; *P* = 0.010), and duration of straight leg raise exercise (*P* = 0.012) were all independent risk factors for residual postoperative pain after PLIF surgery (Table 2).

**Table 2:** Multivariate logistic regression analysis of risk factors related to short-term pain after PLIF surgery.

**Influencing factor**	***B***	**OR**	**95% CI**	***P-*value**
Gender	−0.084	0.919	0.493–1.713	0.791
**SAS**				
1		Reference		
2	0.594	1.812	0.864–3.799	0.116
3	1.194	3.302	0.978–11.146	0.054
4	0.916	2.500	0.359–17.405	0.355
**Surgical segment**				
1		Reference		
2	0.760	2.139	1.099–4.162	0.025
3	1.382	3.981	1.338–11.843	0.013
**Drainage volume**				
< 50 ml		Reference		
≥50 ml	0.936	2.549	1.281–5.072	0.008
PMI	−0.047	0.954	0.840–1.083	0.467
MMI	−1.778	0.169	0.093–0.308	< 0.001^*^
**Average daily standing time**				
< 90 min		Reference		
≥90 min	0.801	2.228	1.212–4.098	0.010
Straight leg raise exercise	−0.645	0.525	0.318–0.865	0.012

SAS, Self-Rating Anxiety Scale; PMI, Psoas Muscle Index; MMI, Multifidus Muscle Index. ^*^Represents P < 0.001.

## 4 Discussion

In this retrospective case-control study comprising 316 patients undergoing PLIF for degenerative lumbar spine disease, we examined a broad spectrum of factors potentially influencing short-term postoperative pain. Our multivariate analysis identified the number of fused segments, drainage volume at the time of drain removal, multifidus muscle index (MMI), average standing time during the first 2 weeks postoperatively, and duration of straight leg raise exercises as independent predictors. Unlike most previous studies that have predominantly focused on surgical technique, anesthetic methods, or the degree of neural decompression, this study integrated biological (MMI), psychological (SAS), behavioral (e.g., early functional exercise times), and surgical parameters, thereby providing a more comprehensive perspective on the mechanisms underlying early postoperative pain.

Our findings underscore that the number of fused segments and the drainage volume at drain removal are independent risk factors. Multiple-level fusion typically entails more extensive soft tissue dissection, wider muscle injury, and increased intraoperative bleeding and exudation. Compared to single-level procedures, multi-level fusions may heighten local inflammatory responses, resulting in tissue edema, fluid accumulation, and an increased likelihood of sustained postoperative pain. Moreover, a higher drainage volume may serve as an indirect indicator of ongoing local tissue injury and exudative responses. Elevated fluid retention and inflammatory mediators can stimulate nociceptors, exacerbating pain (18). Incomplete tissue healing, persistently high drainage volumes, or hematoma formation may further compress neural structures and prolong pain symptoms. In clinical practice, careful patient selection to minimize unnecessary fusion levels, combined with refined surgical techniques and intraoperative hemostatic measures, may reduce tissue trauma. Additionally, patients with higher drainage volumes may benefit from enhanced analgesic, anti-inflammatory, and rehabilitation interventions, and potentially delayed drain removal to mitigate ongoing discomfort.

The robust association between paraspinal muscle health and postoperative pain is also noteworthy. The psoas major and multifidus muscles are key stabilizers of the lumbar spine, modulating posture and load distribution (19, 20). When muscle quality diminishes, fat infiltration increases, or muscle tension declines, spinal stability is compromised. Even minor mechanical stresses may elicit persistent discomfort and pain postoperatively. Surgical manipulation, retractor placement, and soft tissue trauma can further impair muscle blood supply and function, aggravating postoperative pain. Preoperative MRI-based assessments of paraspinal muscles could help stratify patients according to their risk of postoperative pain. In patients identified with poor paraspinal muscle status, targeted core muscle strengthening programs may be introduced preoperatively to enhance muscle quality and function, thereby potentially attenuating postoperative pain and expediting recovery (21, 22).

Our findings also highlight the importance of postoperative rehabilitation, specifically early standing and straight leg raise exercises. Appropriate early mobilization improves blood flow, reduces inflammatory mediator accumulation, and supports tissue healing, collectively alleviating pain and accelerating functional restoration. From a psychological standpoint, early ambulation may reinforce patients' perception of procedural success and alleviate their concerns about pain, thereby potentially modulating pain perception. Postoperative activity should progress gradually. During the initial healing phase, inflammation, oedema, and pain sensitivity persist. Extended standing or walking requires lumbar and back muscles to stabilize the spine, while incompletely healed soft tissues may experience pain due to compression and load-bearing. Bony fusion takes months to form a solid bridge; implants provide early support, but the structure remains fragile. Prolonged standing or walking increases stress on the surgical area, triggering pain (23). Early mobilization (walking and straight leg raises) is currently the consensus for postoperative care following surgery, as it improves clinical outcomes and accelerates the rehabilitation process. This is the recommended exercise regimen after PLIF surgery to alleviate postoperative pain. However, some patients experiencing severe postoperative pain may resist these exercises, while those with milder pain may require a longer rehabilitation period. Our findings merely indicate shorter exercise duration in the pain group, which may reflect reverse causality. Hence, personalized rehabilitation protocols that account for individual muscle status, psychological condition, and wound healing progress are essential. Clinicians and nursing staff should dynamically adjust exercise intensity and duration to prevent exercise-induced exacerbation of pain (24).

Although SAS scores (anxiety levels) were not independent predictors of pain in the multivariate analysis, the significant association observed in univariate analysis does not diminish the potential importance of psychological factors. Anxiety and other negative emotions likely interact with biological and behavioral variables, indirectly influencing pain perception (25, 26). For patients presenting with elevated anxiety, perioperative psychological support, sedation, cognitive-behavioral therapy, or pharmacological intervention could minimize the amplifying effect of negative affect on pain. Future studies should incorporate additional psychological assessments (depression scales, pain catastrophizing scales) as well as evaluations of patients' pain-related beliefs and social support to further elucidate the psychological mechanisms underlying postoperative pain.

Previous studies addressing early postoperative pain following PLIF have often concentrated on isolated factors, such as advancements in surgical techniques (minimally invasive vs. conventional approaches), perioperative analgesic protocols, or demographic variables such as age and BMI (8, 27). By contrast, this study's strength lies in its multifaceted approach, combining biological (muscle indices), psychological (SAS scores), behavioral (postoperative exercise duration), and surgical (fusion segments, drainage volume) factors. Such a holistic strategy is in line with the contemporary precision medicine paradigm, aiming to capture the complexity of clinical presentations through multidimensional data analysis and thus draw conclusions that are closer to real-world clinical scenarios.

By focusing on short-term postoperative pain—a clinically pressing issue—this study contributes to the understanding of factors that, if left unmanaged, may predispose patients to prolonged recovery periods and potentially influence long-term outcomes. Early identification of risk factors allows clinicians and nursing staff to implement preventive measures during the perioperative period. All in all, our findings support the implementation of practical intervention protocols throughout the surgical pathway. Preoperative assessment should incorporate MRI-based paraspinal muscle evaluation and SAS scoring; patients with low MMI scores or elevated SAS scores may benefit from targeted preoperative rehabilitation (core muscle endurance training and hip extensor exercises) and expectation management. During the intraoperative phase, avoid unnecessary multilevel fusions when clinically feasible and prioritize meticulous hemostasis to reduce postoperative drainage burden. Early postoperative care (0–48 h) should employ multimodal analgesia and volume-guided drainage management (record daily drainage volume; consider delayed tube removal if drainage remains persistently high with pain/swelling). Early rehabilitation (days 1–14) should follow a structured, symptom-limited protocol: progressively increase standing time and straight leg raise training, set clear daily limits, and adjust intensity based on pain response.

Nonetheless, several limitations must be acknowledged. As a retrospective study, our findings are subject to potential information bias due to variations in medical records and follow-up completeness. Prospective, longitudinal studies with standardized data collection and extended follow-up intervals are warranted to confirm our results and clarify the interplay between short-term pain and long-term outcomes such as fusion quality and functional restoration. Moreover, future investigations could integrate surgical factors, psychological variables, muscle imaging data, rehabilitation metrics, and social support parameters into a comprehensive dataset. The application of machine learning and artificial intelligence modeling may further refine predictions and identify nuanced, individualized risk profiles for postoperative pain.

This study offers a multifactorial perspective on the determinants of short-term postoperative pain following PLIF. By elucidating the roles of surgical parameters, paraspinal muscle indices, psychological factors, and rehabilitation behaviors, we provide a foundation for more targeted, patient-centered interventions aimed at mitigating early postoperative pain, improving satisfaction, and ultimately enhancing the quality of care for patients with degenerative lumbar spine disease.

## 5 Conclusion

This study, by employing multifactorial analyses, identifies key determinants of short-term postoperative pain following PLIF from multiple dimensions—including the number of fused levels, drainage volume, paraspinal muscle condition, and postoperative rehabilitation exercises. The findings underscore that short-term pain is not merely a consequence of a single surgical technique or individual patient characteristic, but rather a complex phenomenon shaped by multiple interacting factors. The health of deep paraspinal muscles such as the multifidus plays a critical role in maintaining spinal stability and alleviating pain, while appropriate early mobilization and structured rehabilitation exercise prescriptions are equally pivotal. At the same time, surgical strategy, perioperative management, and patient psychological status form a complex network that modulates pain. Identifying these independent risk factors and implementing targeted interventions may enable clinicians to optimize postoperative rehabilitation, enhance patient experiences, and ultimately improve overall treatment quality.

## Data Availability

The raw data supporting the conclusions of this article will be made available by the authors, without undue reservation.

## References

[1] Li H Tang Y Liu Z Chen K Zhang K Hu S . Lumbar instability remodels cartilage endplate to induce intervertebral disc degeneration by recruiting osteoclasts via Hippo-CCL3 signaling. Bone Res. (2024) 12:34. doi: 10.1038/s41413-024-00331-x 38816384 PMC11139958

[2] Sun H Tang W Deng L You X Shen Z Sun X . Development and validation of interpretable machine learning models incorporating Paraspinal muscle quality to predict cage subsidence risk following posterior lumbar interbody fusion. Spine. (2025) 50:1375–85. doi: 10.1097/BRS.0000000000005388 40331716 PMC12456198

[3] Fenton-White HA. Trailblazing: the historical development of the posterior lumbar interbody fusion (PLIF). Spine J. (2021) 21:1528–41. doi: 10.1016/j.spinee.2021.03.016 33757870

[4] Zhang J Fang T You X Tang Y Ruan X Sun H . Role of paraspinal muscle degeneration as a predictor of chronic low back pain in lumbar spondylolisthesis: a machine learning model is developed. Global Spine J. (2025). doi: 10.1177/21925682251383166. [Epub ahead of print]. 41017755 PMC12479446

[5] Yee T Zammar S Mummaneni PV. The lumbar interbody fusion trial: TLIF or PLIF for lumbar spondylolisthesis? Lancet Reg Health Eur. (2024) 43:101000. doi: 10.1016/j.lanepe.2024.101000 39070759 PMC11283005

[6] Plantz MA Hsu WK. Single-level posterolateral fusion (PLF) alone and posterior interbody fusion (PLIF/TLIF) alone lead to a decreased risk of short-term complications compared to combined PLF With PLIF/TLIF procedures: a matched analysis. Spine. (2020) 45:E1391–9. doi: 10.1097/BRS.0000000000003615 32796465

[7] Sun H Tang W You X Deng L Chen L Qian Z . The role of the lumbar paravertebral muscles in the development of short-term residual pain after lumbar fusion surgery. Spine. (2025) 50:537–47. doi: 10.1097/BRS.0000000000005303 39967515 PMC11927452

[8] Capo G Calvanese F Vandenbulcke A Zaed I Di Carlo DT Cao R . Lateral-PLIF for spinal arthrodesis: concept, technique, results, complications, and outcomes. Acta Neurochir. (2024) 166:123. doi: 10.1007/s00701-024-06024-y 38451339

[9] Li X Liu J Liu Z. Comparison of the results of open PLIF versus UBE PLIF in lumbar spinal stenosis: postoperative adjacent segment instability is lesser in UBE. J Orthop Surg Res. (2023) 18:543. doi: 10.1186/s13018-023-04038-3 37516831 PMC10386635

[10] Berg B Gorosito MA Fjeld O Haugerud H Storheim K Solberg TK . Machine learning models for predicting disability and pain following lumbar disc herniation surgery. JAMA Netw Open. (2024) 7:e2355024. doi: 10.1001/jamanetworkopen.2023.55024 38324310 PMC10851101

[11] Stanuszek A Jedrzejek A Gancarczyk-Urlik E Kołodziej I Pisarska-Adamczyk M Milczarek O . Preoperative paraspinal and psoas major muscle atrophy and paraspinal muscle fatty degeneration as factors influencing the results of surgical treatment of lumbar disc disease. Arch Orthop Trauma Surg. (2022) 142:1375–84. doi: 10.1007/s00402-021-03754-x 33484312

[12] Liu S Reitmaier S Mödl L Yang D Zhang T Becker L . Quality of lumbar paraspinal muscles in patients with chronic low back pain and its relationship to pain duration, pain intensity, and quality of life. Eur Radiol. (2025) 35:3652–60. doi: 10.1007/s00330-024-11236-y 39644421 PMC12081590

[13] Lee JM Lee DH Chung NS Chung HW Koh JH Yoon Y . Hip, abdomen, and paraspinal muscle morphologies and their correlation with pain and disability in degenerative lumbar scoliosis patients. Spine. (2025) 50:1589–96. doi: 10.1097/BRS.0000000000005362 40237206

[14] Lin GX Ma YM Xiao YC Xiang D Luo JX Zhang GW Ji ZS . The effect of posterior lumbar dynamic fixation and intervertebral fusion on paraspinal muscles. BMC Musculoskelet Disord. (2021) 22:1049. doi: 10.1186/s12891-021-04943-w 34930199 PMC8690627

[15] Tekin ZN Karatekin BD. Relationship between the properties of paraspinal muscles and bone mineral density in osteoporotic patients. J Coll Physicians Surg Pak. (2022) 32:1137–42. doi: 10.29271/jcpsp.2022.09.1137 36089709

[16] Rezazadeh F Taheri N Okhravi SM Hosseini SM. The relationship between cross-sectional area of multifidus muscle and disability index in patients with chronic non-specific low back pain. Musculoskelet Sci Pract. (2019) 42:1–5. doi: 10.1016/j.msksp.2019.03.005 30981101

[17] Dunstan DA Scott N. Norms for Zung's Self-rating anxiety scale. BMC Psychiatry. (2020) 20:90. doi: 10.1186/s12888-019-2427-6 32111187 PMC7048044

[18] Reier L Fowler JB Arshad M Siddiqi J. Drains in spine surgery for degenerative disc diseases: a literature review to determine its usage. Cureus. (2022) 14:e23129. doi: 10.7759/cureus.23129 35464540 PMC9001810

[19] Hildebrandt M Fankhauser G Meichtry A Luomajoki H. Correlation between lumbar dysfunction and fat infiltration in lumbar multifidus muscles in patients with low back pain. BMC Musculoskelet Disord. (2017) 18:12. doi: 10.1186/s12891-016-1376-1 28068962 PMC5223418

[20] Zhong Y Liu J Zhou W Yu D. Relationship between straight leg-raising test measurements and area of fat infiltration in multifidus muscles in patients with lumbar disc hernation. J Back Musculoskelet Rehabil. (2020) 33:57–63. doi: 10.3233/BMR-181304 31006661

[21] Qiao G Feng M Liu J Wang X Ge M Yang B . Does the position of cage affect the clinical outcome of lateral interbody fusion in lumbar spinal stenosis? Global Spine J. (2022) 12:204–8. doi: 10.1177/2192568220948029 32856471 PMC8907639

[22] Nan C Ma Z Liu Y Ma L Li J Zhang W. Impact of cage position on biomechanical performance of stand-alone lateral lumbar interbody fusion: a finite element analysis. BMC Musculoskelet Disord. (2022) 23:920. doi: 10.1186/s12891-022-05873-x 36258213 PMC9578219

[23] Coronado RA Master H White DK Pennings JS Bird ML Devin CJ . Early postoperative physical activity and function: a descriptive case series study of 53 patients after lumbar spine surgery. BMC Musculoskelet Disord. (2020) 21:783. doi: 10.1186/s12891-020-03816-y 33246446 PMC7697379

[24] Huang J Li P Wang H Lv C Han J Lu X. Exploring elderly patients' experiences and concerns about early mobilization implemented in postoperative care following lumbar spinal surgery: a qualitative study. BMC Nurs. (2023) 22:355. doi: 10.1186/s12912-023-01510-7 37794348 PMC10552231

[25] Rajjoub R Sammak SE Rajjo T Rajjoub NS Hasan B Saadi S . Meditation for perioperative pain and anxiety: a systematic review. Brain Behav. (2024) 14:e3640. doi: 10.1002/brb3.3640 39073307 PMC11284642

[26] Sharif-Nia H Nazari R Hajihosseini F Froelicher ES Osborne JW Taebbi S . The relationship of fear of pain, pain anxiety, and fear-avoidance beliefs with perceived stress in surgical patients with postoperative kinesiophobia. BMC Psychol. (2025) 13:420. doi: 10.1186/s40359-025-02743-8 40264201 PMC12016382

[27] Mehren C Ostendorff N Schmeiser G Papavero L Kothe R. Do TLIF and PLIF techniques differ in perioperative complications? - Comparison of complications rates of two high volume centers. Global Spine J. (2025) 15:84–93. doi: 10.1177/21925682241248095 38631328 PMC11572157

